# Systematic Review and Meta-Analysis of Prevalence of Coeliac Disease in Women with Infertility

**DOI:** 10.3390/nu11081950

**Published:** 2019-08-20

**Authors:** Mercedes Castaño, Rubén Gómez-Gordo, David Cuevas, Concepción Núñez

**Affiliations:** 1 Laboratorio de investigación en Genética de enfermedades complejas, Hospital Clínico San Carlos, IdISSC, 28040 Madrid, Spain; 2 Laboratorio de Biología Vascular y Microbiota, Hospital Clínico San Carlos, IdISSC, 28040 Madrid, Spain

**Keywords:** coeliac disease, reproductive disorders, infertility, recurrent abortion

## Abstract

We aimed to estimate the seroprevalence and the prevalence of coeliac disease (CD) in women with reproductive problems. A systematic review of English published articles until June 2019 was performed in PubMed and Scopus using the terms: (infertility and (coeliac disease OR gluten) OR (miscarriage and (coeliac disease OR gluten) OR (abortion and (coeliac disease OR gluten). All articles showing numerical data of anti-transglutaminase type 2 or anti-endomisium antibodies, or intestinal biopsy information were included. The study group comprised women with overall infertility, unexplained infertility, or recurrent spontaneous abortions. Two authors independently performed data extraction using a predefined data sheet. The initial search yielded 310 articles, and 23 were selected for data extraction. After meta-analysis, the pooled seroprevalence was very similar for overall and unexplained infertility, with a pooled proportion of around 1.3%–1.6%. This implies three times higher odds of having CD in infertility when compared to controls. The pooled prevalence could not be accurately calculated due to the small sample sizes. Further studies with increased sample sizes are necessary before giving specific recommendations for CD screening in women with reproductive problems, but current data seem to support a higher risk of CD in these women.

## 1. Introduction

Coeliac disease (CD) is a systemic autoimmune disorder causing enteropathy, which occurs after ingestion of dietary gluten in genetically susceptible individuals. It shows a worldwide prevalence of approximately 1%, although this value can differ depending on age, sex, and geographic location [1]. Classically associated with gastrointestinal symptoms, the clinical manifestations of CD are very heterogeneous and also include non-gastrointestinal symptoms such as infertility. The first connection between infertility and CD was described by Morris et al. in 1970 [2], who also explained the reversion of infertility after a gluten-free diet (GFD) in three CD women. In recent years, the interest in gynecologic and obstetric manifestations of CD has been increasing. Diverse studies have associated CD with numerous bad reproductive outcomes [3,4,5], and a debate exists about including these women as a risk group for CD testing. Three population-based studies failed to find greater likelihood of fertility problems in women with CD [6,7,8], although one of them described decreased fertility in the two years preceding CD diagnosis [8]. Regarding the prevalence of CD, contradictory results have been reported [9,10], with some works describing increased prevalence of CD in women with infertility [11,12], but others showing around the 1% accepted in the general population [13,14]. In an attempt to solve this, some meta-analyses have been performed, but important issues such as the different diagnostic work-up followed, the definition of infertility, the ethnicity or the use of appropriate sample sizes have not been taken into account. As a matter of fact, some works based their diagnoses on serological tests that can differ among studies, and others did so on the confirmatory biopsy. However, these limitations are not usually considered in the published papers or meta-analyses. Therefore, despite it being commonly accepted that CD may have implications on women’s reproductive health, there is not a consensus about its relevance and CD testing is not recommended in women with bad reproductive outcomes in clinical practice.

It must be considered that a lifelong gluten-free diet is expected to prevent complications in pregnancy for CD patients. This, combined with the emotional and economic impact of infertility, makes necessary to revise the current knowledge about infertility and CD in order to know the expected impact and real implications. Hence, we aimed to perform a systematic review and a meta-analysis of the published studies related to CD and bad reproductive outcomes, to estimate the pooled prevalence and seroprevalence of CD in women with overall infertility, unexplained infertility, and recurrent spontaneous abortions (RSA).

## 2. Material and Methods

We performed a systematic review and a meta-analysis to know the prevalence of undiagnosed CD and the seroprevalence of CD in women with reproductive problems. Subgroup analyses were performed in order to eliminate a possible source of bias. Three groups of women were considered according to the presentation of overall infertility, unexplained infertility or RSA. The group of overall infertility comprises reproductive problems due to any cause (including known causes) and includes unexplained infertility and RSA. Unexplained infertility refers to infertility in which no cause was identified after excluding the most frequent causes with previous screening tests. RSA is defined by the presence of a specific number of consecutive spontaneous abortions.

Systematic review and meta-analysis were performed adhering to PRISMA guidelines [15]. They were independently performed by two authors (C.N. and M.C.) and disagreements were solved after discussion.

### 2.1. Information Sources

The electronic search was performed in PubMed and in Scopus up to 31 May 2019. The terms of search were: (infertility and (coeliac disease OR gluten) OR (miscarriage and (coeliac disease OR gluten) OR (abortion and (coeliac disease OR gluten). The articles were collected in an EndNote library.

### 2.2. Eligibility Criteria

The following eligibility criteria were considered: (1) to include women with infertility problems, unexplained infertility or RSA as subjects of study; and (2) to show numerical data of anti-transglutaminase type 2 (TG2) or anti-endomisium (EMA) antibody tests used in CD screening and/or results of duodenal biopsy. Only articles published in English were considered. No contact with the authors to request additional information was performed in any case.

Risk of bias in individual studies was not used to obtain the final selection of articles. The wide controversy related to CD and infertility relies on all the published work and we aimed to show the basis of such a controversy.

### 2.3. Synthesis of Results

For data extraction, an Excel file was created in which both authors independently recorded information related to the variables included in Table 1, Table 2 and Table 3. In Table 1, we included information about first author, year of publication, ethnicity or country of origin of the studied group and characteristics of the women (with definition of infertility and RSA). Ethnicity was filled considering the country of study. In countries with several ethnic groups in relevant proportions, specifications were added when that information was present in the original paper. Table 2 included summary information about screening tests for CD, criteria used for CD diagnosis and characteristics of the control group. Information about total IgA measure, relevant in order to discard IgA deficiency, disorder that shows increased prevalence in CD and causes negative IgA-based serology, was also added. In Table 3, we included the extracted data used to carry out meta-analysis studies: sample size, number of women with positive IgA anti-TG2 antibodies, number of women with positive IgA EMA and number of women with CD compatible biopsy. With these data, we attempted to address the three objectives of the study: (1) to know the seroprevalence of CD, commonly estimated by IgA anti-TG2; (2) to know the seroprevalence of CD estimated by IgA EMA, which presents higher specificity; (3) to know the prevalence of CD. 

### 2.4. Statistical Analysis

Meta-analyses were performed using the meta package [16] in the software R. Proportions were transformed using the Freeman-Tukey double arcsine transformation to calculate an overall proportion. The DerSimonian-Laird estimate was used in the random effects model to estimate the between-study variance. We also analyzed case-control data using the Odds Ratio (OR) with a 95% confidence interval (95% CI), and using the Mantel-Haenszel method to calculate the fixed effects estimate.

Heterogeneity between studies was assessed by the I^2^ statistic and the Cochran Q test. I^2^ values below 25% were considered no heterogeneity, and up to 40% might not be important heterogeneity. Cochran p values below 0.1 were considered significant. Funnel plots were performed in order to graphically assess potential publication bias, which was statistically evaluated with the Egger’s test. Overall proportions were expressed using the fixed model for I^2^ < 25%, the random model was chosen for higher I^2^ values. 

Sensitivity analyses were performed in order to explore the influence of individual studies in the main findings of the meta-analysis. Different criteria such as sample size and ethnicity were used. 

In addition to the three groups of women analyzed (overall infertility, unexplained infertility and RSA), other three subsets were established based on diagnostic criteria: anti-TG2, EMA, and biopsy results.

## 3. Results

### 3.1. Study Selection

**Figure 1:**
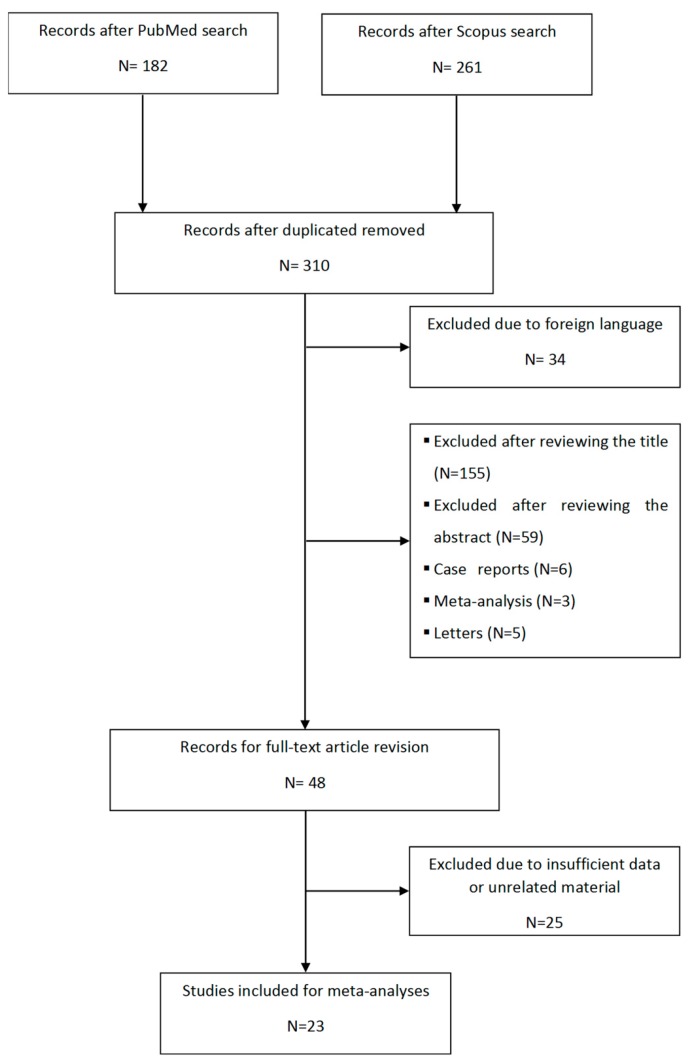
Flowchart outlining the study selection process in the systematic review and meta-analysis.

The electronic search resulted in 182 records in PubMed and 261 in Scopus. All articles were downloaded to EndNote and duplicate records were excluded, obtaining a total of 310 articles. After review, 34 were excluded by language because they were not published in English, 214 were eliminated based on the information provided in their title and/or abstract and 14 were eliminated because they only included letters, case reports, or meta-analyses. A total of 48 articles were selected for full-text revision. Out of these articles, 25 papers were eliminated given that they showed insufficient data or unrelated material. Finally, 23 studies were selected. In addition, new articles were searched in the bibliographic references of the selected articles, but no additional studies were found. Figure 1 shows the flowchart used.

### 3.2. Characteristics of the Included Studies.

Table 1 contains information about the 23 articles that were finally selected for the study. All of them were considered for studying overall infertility, 16 for unexplained infertility and 7 for RSA. Infertility was defined by some authors as the failure to conceive after at least 12 months of unprotected intercourse, reduced to 6 months for women aged 35 and older in one paper [29]. However, in some cases, authors talk about infertile women but a definition is not included or they consider them as those attending to fertility clinics or undergoing assisted reproductive technology. Unexplained infertility was commonly defined as the infertility present when there is no apparent cause for infertility after review of medical history, physical examination, and specific tests. In women, these tests are focused on ovarian reserve, ovulatory function and structural abnormalities, but they can differ among studies; in males, screening tests are focused on semen analysis. Some authors only indicate that women with unexplained infertility are included, adding no information. RSA is considered in most papers when suffering ≥2 consecutive spontaneous abortions, although two authors define it with ≥3 abortions [18,21]. All works consider only unexplained RSA but Collin et al. [9], who do not provide that information, and Kutteh et al. [33], who consider RSA of known and unknown cause. 

The type of study in all cases but one [23] was prospective. 

European populations were studied in 8 works [9,10,14,17,18,19,23,32], and 8 additional studies consider populations with a predominant Caucasian component [12,13,21,22,25,30,31,33]. The remaining 7 studies include populations from countries with an important representation of different ethnic groups [11,20,24,26,27,28,29].

Regarding CD, Table 2 shows information about the screening tests and the criteria used for CD diagnosis. The definition of CD differs among authors, sometimes including only seroprevalence. Specifically, 10 works were based only on serological results [10,11,18,21,23,25,28,30,31,33] and 13 included a duodenal biopsy after serological screening [9,12,13,14,17,19,20,22,24,26,27,29,32]. It must be also considered that the selected articles were published between 1976 and 2019, and the serological tests used for CD screening vary according to the technological and scientific advances. First studies included anti-reticulin and anti-gliadin antibodies, adding or being replaced by the more specific EMA and anti-TG2 antibodies in most recent papers. This is relevant for papers only including seroprevalence, but also for those using serological tests previous to the biopsy. In some works, total IgA is evaluated in order to identify patients with IgA deficiency. It is noteworthy that only one study uses HLA to support diagnosis [12].

Data of controls to be used as a reference for general prevalence or seroprevalence in the same geographical region are considered in 20 works (Table 2). In 12 cases, a sample of healthy fertile women is used as a control group to test CD, with slight differences among studies. However, other works consider the general prevalence in the country or region, usually based on adult data but Meloni et al. [19] who used school children previously screened for CD as controls.

A common characteristic to most of studies is the low sample size used (Table 3). There are only seven papers [10,11,23,30,31,32,33] with more than 300 women studied due to overall infertility (range 45–995, mean 258 ± 50.7), and those numbers are even reduced when considering unexplained infertility (range 25–351, mean 123.9 ± 24.3) and recurrent miscarriage (range 45–708, mean 172 ± 90.1).

### 3.3. Meta-Analysis

Three meta-analyses were performed, focused on overall infertility, unexplained infertility, and RSA (Table 4).

#### 3.3.1. Overall Infertility

- **Anti-TG2 data**

**Figure 2:**
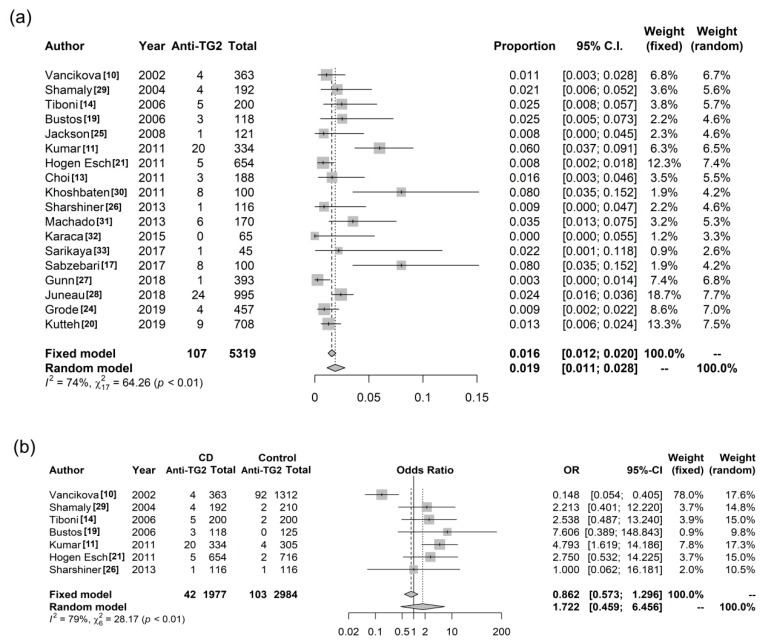
Forest plot based on anti-TG2 data of (**a**) pooled seroprevalence; (**b**) pooled odds ratio of coeliac disease in women with overall infertility.

Eighteen studies [10,11,13,14,20,21,22,23,24,25,26,27,28,29,30,31,32,33] reported the seroprevalence of CD assessing anti-TG2 antibodies in women presenting overall infertility, comprising a total of 5319 women. Figure 2a shows the forest plot with the results of this meta-analysis. The pooled seroprevalence using a random model was 1.9% (95% CI 1.1–2.8%). However, very high heterogeneity was observed: I^2^ = 74%, *p* Cochran < 0.0001; with significant bias of publication (*p* Egger = 0.0046). Removal of any single study reduced heterogeneity below 65%. When looking at the four studies giving the most extreme proportions (highest or lowest), three of them corresponded to studies with sample sizes ≤100. Thus, only works with N > 100 were subsequently analyzed, but heterogeneity remained high (69%) although with lower bias (*p* Egger = 0.018) and the pooled proportion decreased to 1.6% (95% CI 1.0–2.4). No obvious source of heterogeneity was found. Sensitive analyses were performed classifying studies depending on they include predominantly Caucasian [10,13,14,21,22,23,25,30,31,32,33] or non-Caucasian [11,20] populations. Heterogeneity decreased to 43% in the Caucasian group (pooled proportion = 1.2 95% CI 0.7–1.7) and remained very high (I^2^ = 78%, *p* Cochran = 0.0322) in the group of non-Caucasian populations (pooled proportion = 3.9 95% CI 1.0–8.5), although only two studies comprising a total of 526 women were included. 

When considering a control arm (Figure 2b), high heterogeneity (I^2^ = 79%, *p* Cochran < 0.0001) was also present initially, but one study [10] reported a surprisingly high number of control individuals with anti-TG2 antibodies (92 out of 1312 subjects), notably when compared to the low number (6) of those also presenting EMA. After excluding it, heterogeneity disappeared (I^2^ = 0%, *p* Cochran = 0.87) and increased seroprevalence of CD was observed in women with infertility: OR = 3.4 (Table 4).

The pooled seroprevalence measured with anti-TG2 in 1672 controls was 0.5% 95% CI 0.2–1.1 (I^2^ = 13, *p* Cochran = 0.33) after excluding the study of Vancikova et al. [10] (otherwise I^2^ = 94%, *p* Cochran < 0.0001). 

Heterogeneity does not seem to be due to the Caucasian or non-Caucasian origin and thus this factor was not considered further.

- **EMA data**

A total of 12 studies [10,11,13,14,18,20,22,23,25,26,31,33] assessing CD seroprevalence with EMA remained after excluding 4 with N ≤ 100, which comprise a total of 4233 women. High heterogeneity was also present after meta-analysis: I^2^ = 65%, *p* Cochran = 0.0009, leading to a pooled frequency of 1.3% 95% CI 0.7–2.1%.

In controls, the pooled seroprevalence of CD based on EMA data of 2859 individuals was 0.3 (95% CI 0.1–0.6) (I^2^ = 0, *p* Cochran = 0.54). The case-control meta-analysis showed again no heterogeneity and a significantly increased risk of CD in infertility: OR = 3.0.

- **Biopsy data**

Six studies with N > 100 [9,13,14,20,26,32] reported the prevalence of CD based on compatible duodenal biopsy in women with overall infertility. A total of 1407 women were enrolled in this meta-analysis, which showed a pooled prevalence of 1.5% 95% CI 0.6%–2.8% with moderate heterogeneity.

Meta-analysis of controls showed a pooled CD prevalence of 0.4 95% CI 0–1.3 (I^2^ = 0, *p* Cochran = 0.43). The case-control meta-analysis gave a significantly increased prevalence in infertile women: OR = 4.1.

#### 3.3.2. Unknown Infertility

CD seroprevalence was 1.3%–1.5%, considering EMA or anti-TG2, respectively. Compared to controls, these values imply a three-fold risk in women with infertility (Table 4). 

Only seroprevalence could be estimated, since there is only one study reporting biopsy data with N > 100 [20]: OR = 5.5 95% CI 0.6–255.5, but it only includes 192 infertile women and 200 controls. 

#### 3.3.3. RSA

There are not studies with biopsy performed and N > 100, allowing only seroprevalence calculation again. In this case, different values were observed when considering anti-TG2 and EMA, 2.2% and 1.1%, respectively, with wide confidence intervals (Table 4), but the sample size of all studies but one was lower than 120 women.

## 4. Discussion

The present work represents the most complete systematic review and meta-analysis performed in relation to CD and infertility. The full text of 23 papers was revised attempting to identify the characteristics of the published works and to achieve some conclusive data. Since 1976, when the first work studying CD prevalence in infertile women dates from reference [17], numerous works have tried to shed some light on this subject. However, the contradictory findings maintain this issue as a matter of debate.

The lack of conception after regular intercourse for 12 months qualifies couples for assisted reproductive technology. Women with infertility and CD may be overtreated. In addition, treatment can extend over long periods because undetected CD can also hinder the success of the reproductive treatment. In those cases, natural conception could be achieved under a gluten free diet.

In this work, we try to focus the attention on the different points that may be underlying the reported discrepancies. First of all, we consider the characteristics of the studied women. Three subgroups of study were established: overall infertility, unexplained infertility, and RSA. Overall infertility involves women with reproductive problems of known cause, but also the other two groups, since it is frequent for women attending to a fertility clinic and/or undergoing assisted reproductive technology to be studied. In most of these women, the cause of infertility remains unknown and they can visit the clinic due to lack of conception for a long period but also for recurrent abortions. We find CD seroprevalence to be very similar among the groups of women with overall and unexplained infertility (Table 4). RSA cannot be properly evaluated due to the low sample sizes, but CD seroprevalence may be only slightly higher. Thus, the group of study does not seem to account for the previous lack of uniform results. 

Other potential source of discrepancy could come from the diagnostic work-up. Some studies assessed prevalence only by serology. We consider prevalence and seroprevalence independently. In this regard, there are also important differences, with some authors using the most sensitive and specific antibody combination IgA anti-TG2/EMA, but others considering CD also when present IgA/G anti-gliadin or anti-PDG antibodies (Table 2). To avoid the differences that can emerge due to divergence in the diagnostic accuracy of the tested antibodies, we collected data corresponding only to anti-TG2 and EMA. The pooled seroprevalence obtained with both antibodies is very similar, only slightly higher when looking at anti-TG2 antibodies, as expected [1]. These values are around 1.3-1.6%, but the meta-analyses showed high heterogeneity among studies (I^2^ > 60%). This heterogeneity remained high when stratifying by a predominantly Caucasian or not Caucasian origin of the included populations, but it disappeared when data of infertile women were meta-analyzed including control groups. These case-control meta-analyses showed homogeneity and revealed approximately three times higher seroprevalence of CD in women with overall and unexplained infertility. It is well-known that CD seroprevalence depends on the geographical region [1,34] and this hampers to know if some value is high or low when a reference value in that specific population is not given. Prevalence varies also according to the sex and age, thus a proper selection of the control group is very important. For case-control meta-analysis of seroprevalence, we used studies comprising adult women as controls, in most cases with proven fertility. The work of Meloni et al. [19], which compares CD data in infertile women with the prevalence obtained in school children was not included in calculations. Concordantly, the individual meta-analyses of controls showed low values of CD seroprevalence, around 0.3–0.5%.

It has been largely discussed that the screening of CD in infertility does not deserve attention because prevalence in this group is thought to be similar to the one obtained in the general population. This idea was predominantly based on the 1% seroprevalence observed in several works, which was assumed as similar to the worldwide CD seroprevalence. As earlier explained, CD prevalence differs depending of the location, but also on sex and age. The value of 1% corresponds to the seroprevalence including children, but around half of seroprevalence is expected in adults than children [1]. This is evidenced when controls are evaluated. 

Sample sizes also constitute a source of heterogeneity. As previously observed [34], the studies with the smallest sample sizes tended to produce both the lowest and the highest values of seroprevalence. The wide confidence intervals resultant from the meta-analyses we performed are probably a consequence of the small sample sizes. We establish the cut-off in 100 individuals, otherwise too many studies would be excluded. Nevertheless, we are aware that more accurate values would be obtained with higher sample sizes in all studies. Considering a seroprevalence in general adult population = 0.5%, one individual study would need a sample size of approximately 1500 infertile women and 1500 controls to detect an OR = 3 with 80% of statistical power. Therefore, the main limitation of our meta-analyses depends on the limitations of the previous studies, most of which included small sample sizes. 

The lack of a control group is also an important impediment. Other limitations such as variations in CD definition, the diagnostic work-up or the diverse serological tests used for CD screening could be somehow solved with the approaches we followed. Other factors to be considered are those related to the patient selection process that expose studies to selection bias and can contribute to an overestimation or underestimation of the prevalence. Choi et al. [13] developed a non-consecutive voluntary screening. This could overestimate the real prevalence because patients with clinical symptoms or those with relatives with CD are probably willing to participate, but infertile patients with unrecognized CD are often asymptomatic and this may hamper their participation in the study. However, the seroprevalence found by those authors is similar to the pooled value after meta-analysis. The opposite situation could exist in other works, with authors excluding women from risk groups, such as those showing selective IgA deficiency [30] or other associated conditions such as hypothyroidism, diabetes and antiphospholipid antibody syndrome [11]. In these cases, the prevalence could be underestimated, this does not seem to occur in the study of Kumar et al., but a very low seroprevalence is found by Gunn et al.

Regarding CD prevalence, biopsy data were used to calculate a pooled value. Women underwent biopsy in very few studies and prevalence could be only calculated for overall infertility. Nevertheless, the total number of women is very low, around 1400 women for overall prevalence and 600 for the case-control comparisons, and the values obtained cannot be very accurate, especially the OR obtained in the case-control meta-analysis, which shows a very wide confidence interval and is higher than the seroprevalence, contrary to expectations.

## 5. Conclusions

A common feature of the great majority of studies evaluating CD prevalence in women with infertility is the low sample size studied. Seroprevalence based on anti-TG2 and EMA data shows a three times higher risk in women with overall and unexplained infertility than in healthy women. Furthermore, there could be some cases of seronegative CD. An accurate value for prevalence could not be achieved, but considering the values of seroprevalence, higher risk of CD should be expected in infertile women. Thus, these meta-analyses open again the debate about supporting routine screening for CD among infertile patients. It is of utmost importance to make CD women aware of the potential positive impact in adoption of a strict GFD on fertility.

## Figures and Tables

**Table 1:** Selected studies for systematic review: ethnicity and women of study.

First Author	Year of Publication	Ethnicity	Women of Study
Wilson **[17]**	1976	United Kingdom	Primary or secondary infertility without overt disease
Collin **[9]**	1996	Finland	Primary or secondary infertility, unexplained infertility and RSA (≥2 spontaneous abortions)
Kolho **[18]**	1999	Finland	Infertility, unexplained infertility and unexplained RSA (≥ 3 consecutive abortions)
Meloni **[19]**	1999	Italy (Sardinia)	Infertility and unexplained infertility
Vancikova **[10]**	2002	Czech	Immunological infertility ^1^ and unexplained infertility
Fasano **[12]**	2003	US (mainly Caucasian)	Unexplained infertility
Shamaly **[20]**	2004	Arab	Unexplained infertility
Tiboni **[14]**	2006	Italy	Women undergoing assisted reproductive technology with known and unknown cause
Bustos **[21]**	2006	Argentine (Caucasian)	Unexplained RSA (≥3 consecutive spontaneous abortions and no previous live birth)
Jackson **[22]**	2008	US (64% Caucasian, 28% Asian)	Women attending a clinic for reproductive health with unknown cause
Kumar **[11]**	2011	India	Infertility, unexplained infertility and unexplained RSA (≥2 consecutive abortions before week 20)
Hogen Esch **[23]**	2011	The Netherlands (Caucasian)	Women attending a fertility clinic with known and unknown cause
Choi **[13]**	2011	US (63% Caucasian, 15% Asian)	Primary or secondary infertility lasting ≥12 months of duration with known and unknown cause. Voluntary, non-consecutive screening
Khoshbaten **[24]**	2011	Iran	Infertile couples referred to an Infertility Department with unknown cause
Sharshiner **[25]**	2013	US (77% Caucasian)	Unexplained RSA (≥2 consecutive abortions before week 20 and ≤1 previous live birth)
Machado **[26]**	2013	Brazil	Infertility and unexplained infertility lasting ≥12 months
Karaca **[27]**	2015	Turkey	Women undergoing assisted reproductive technology with unknown cause
Sarikaya **[28]**	2017	Turkey	Unexplained RSA (≥2 consecutive spontaneous abortions before week 20)
Sabzebari **[29]**	2017	Iran	Unexplained infertility lasting ≥12 months or ≥6 months in women aged ≥ 35
Gunn **[30]**	2018	Canada (66% Caucasian, 17% Asian)	Infertility (failure to conceive after 12 months of unprotected intercourse) of known and unknown cause
Juneau **[31]**	2018	US	Infertile women undergoing in vitro fertilization
Grode **[32]**	2019	Denmark	Population referred to fertility treatment
Kutteh **[33]**	2019	US (72% Caucasian, 18% African American)	RSA (≥2 clinical abortions) of known and unknown cause

RSA: recurrent spontaneous abortion. ^1^ Inmunological infertility included positive anti-phospholipid, anti-spermatozoal or anti-zona pellucida antibodies.

**Table 2:** Selected studies for systematic review: coeliac disease screening tests and diagnosis, and characteristics of the control group.

First Author	CD Screening Tests	CD Diagnostic Criteria	Control Group
Wilson **[17]**	Anti-reticulin antibodies and biopsy	Biopsy when positive ant-reticulin antibodies and response to the GFD	No controls
Collin **[9]**	IgA anti-gliadin or IgA anti-reticulin antibodies and biopsy	Biopsy when positive anti-gliadin or anti-reticulin antibodies	Women with normal obstetric history who had undergone laparoscopic sterilization
Kolho **[18]**	IgA EMA	Positive EMA	Women personnel without infertility problems (mean number of children 1.5)
Meloni **[19]**	IgA/G anti-gliadin, IgA EMA, total IgA and biopsy	Biopsy when positive 2 out of the 3 antibodies	Healthy school children from the same geographical area
Vancikova **[10]**	IgA/G anti-gliadin, IgA anti-TG2, IgA EMA and total IgA	EMA when positive anti-gliadin or anti-TG2	Healthy blood donors
Fasano **[12]**	IgA/G anti-gliadin, IgA EMA, total IgA, HLA-DQ and biopsy	Positive EMA with biopsy or with compatible HLA	Female not-at-risk subjects
Shamaly **[20]**	IgA anti-TG2, IgA EMA, total IgA and biopsy	Biopsy when positive anti-TG2 or EMA or when IgAD is present	Healthy Arab women
Tiboni **[14]**	IgA anti-TG2, IgA EMA and biopsy	Biopsy when positive anti-TG2 or EMA	Healthy women not reporting reproductive problems with at least one child delivered
Bustos **[21]**	IgA/G anti-gliadin, IgA anti-TG2 and total IgA	Positive 2 out of the 3 antibodies	Argentine Caucasian women of the blood bank with at least two children and without pregnancy losses
Jackson **[22]**	IgA anti-TG2, IgA EMA and biopsy	Positive anti-TG2 and EMA	Prevalence in general population of US (0.8%)
Kumar **[11]**	IgA/G anti-gliadin, IgA anti-TG2 and IgA EMA	Positive anti-TG2	Women with normal obstetric history
Hogen Esch **[23]**	IgA anti-TG2 and IgA EMA	Positive anti-TG2 and EMA	Prevalence in general adult female population of The Netherlands
Choi **[13]**	IgA/G anti-gliadin, IgA anti-TG2, IgA EMA, total IgA and biopsy	Biopsy when positive anti-TG2 or EMA	CD expected in women of similar age in the same geographical area (1.3%)
Khoshbaten **[24]**	IgA/G anti-TG2, total IgA and biopsy	Biopsy when positive anti-TG2	Healthy couples lacking reproductive problems with at least one child delivered
Sharshiner **[25]**	IgA/G anti-TG2 and IgA/G EMA	Positive anti-TG2 or EMA	Healthy women with a history of ≥2 uncomplicated term live births, no more than one pregnancy loss prior week 20, and no major medical or obstetric problems or clinical features of CD
Machado **[26]**	IgA anti-TG2, IgA EMA, total IgA, HLA-DQ and biopsy	Biopsy when positive anti-TG2 or EMA	Prevalence in blood donors in Brazilian regions (0.2–0.5%)
Karaca **[27]**	IgA/G anti-gliadin, IgA/G anti-TG2, IgA/G EMA, total IgA and biopsy	Biopsy when positive anti-gliadin, anti-TG2 or EMA	No controls
Sarikaya **[28]**	IgA/G anti-TG2	Positive anti-TG2	Healthy fertile females with no history of RPL with at least two child delivered
Sabzebari **[29]**	IgA/G anti-TG2, total IgA and biopsy	Biopsy when positive anti-TG2	No controls
Gunn **[30]**	IgA anti-TG2 and total IgA	Positive anti-TG2	No controls
Juneau **[31]**	IgA anti-TG2 and IgA EMA	Positive anti-TG2 or EMA	No controls
Grode **[32]**	IgA anti-TG2, total IgA (IgG anti-PDG when IgA deficiency) and biopsy	Biopsy when positive anti-TG2 or anti-PDG	Prevalence of Danish general population (0.48%)
Kutteh **[33]**	IgA anti-TG2, IgA EMA and IgA anti-PDG	Positive anti-TG2, EMA or anti-PDG	Healthy non-pregnant women with at least one live birth and no miscarriages

TG2: type 2 transglutaminase; EMA: antiendomisium antibodies.

**Table 3:** Data extracted from the selected studies for meta-analysis. The total number of patients analyzed and those with compatible antibody tests or biopsy are shown.

First Author	Infertility				Unexplained Infertility				Recurrent Miscarriage				Control Group			
	Total N	Anti-TG2	EMA	Biopsy	Total N	Anti-TG2	EMA	Biopsy	Total N	Anti-TG2	EMA	Biopsy	Total N	Anti-TG2	EMA	Biopsy
**Wilson [17]**	77	-	-	2	-	-	-	-	-	-	-	-	-	-	-	-
**Collin [9]**	200	-	-	4	98	-	-	4	50	-	-	0	150	-	-	0
**Kolho [18]**	192	-	2	-	47	-	1	-	63	-	1	-	51	-	1	1
**Meloni [19]**	99	-	4	3	25	-	2	1	-	-	-	-	1607	-	-	17
**Vancikova [10]**	363	4	1	-	275	3	0	-	-	-	-	-	1312	92	6	-
**Fasano [12]**	48	-	3	-	48	-	3	-	-	-	-	-	2069	-	15	-
**Shamaly [20]**	192	4	5	5	192	4	5	5	-	-	-	-	210	2	0	1
**Tiboni [14]**	200	5	3	5	53	0	0	-	-	-	-	-	200	2	1	2
**Bustos [21]**	118	3	-	-		-	-	-	118	3	-	-	125	0	-	-
**Jackson [22]**	121	1	1	-	121	1	1	-	-	-	-	-	-	-	-	-
**Kumar [11]**	334	20	16	-	230	13	11	-	104	7	5	-	305	4	3	-
**Hogen Esch [23]**	654	5	5	-	351	2	2	-	-	-	-	-	716	2	2	-
**Choi [13]**	188	3	3	4	51	2	2	3	-	-	-	-	-	-	-	-
**Khoshbaten [24]**	100	8	-	3 *	100	8	-	3 *	-	-	-	-	200	4	-	0
**Sharshiner [25]**	116	1	0	-	-	-	-	-	116	1	0	-	116	1	1	-
**Machado [26]**	170	6	3	2 **	29	3	3	2 **	-	-	-	-	-	-	-	-
**Karaca [27]**	65	0	0	0	65	0	0	0	-	-	-	-	-	-	-	-
**Sarikaya [28]**	45	1	-	-	-	-	-	-	45	1	-	-	41	3	-	-
**Sabzebari [29]**	100	8	-	7	100	8	-	7	-	-	-	-	-	-	-	-
**Gunn [30]**	393	1	-	-	197	1	-	-	-	-	-	-	-	-	-	-
**Juneau [31]**	995	24	22	-	-	-	-	-	-	-	-	-	-	-	-	-
**Grode [32]**	457	4	-	1	-	-	-	-	-	-	-	-	-	-	-	-
**Kutteh [33]**	708	9	6	-	-	-	-	-	708	9	6	-	100	1	1	-

* Biopsy performed in 3 anti-TG2 positive women; ** biopsy performed in 5 seropositive women.

**Table 4:** Summary of the seroprevalence and prevalence of coeliac disease in the different groups of infertile women considered.

Group of Study	Meta-Analysis Proportion			Meta-Analysis Case-Control			
	I^2^	Proportion (95% CI)	N	I^2^	OR (95% CI)	*p*	N infertility:N controls
**Overall infertility**							
Anti-TG2	69	1.6 (1.0–2.4)	5009	0 *	3.4 (1.7–6.6)	0.0002	1614:1672
EMA	65	1.3 (0.7–2.1)	4233	11	3.0 (1.5–5.9)	0.0024	1859:2859
Biopsy	60	1.5 (0.6–2.8)	1407	0	4.1 (1.3–13.2)	0.0229	592:560
**Idiopathic infertility**							
Anti-TG2	71	1.5 (0.4–3.0)	1366	0 *	3.3 (1.4–7.7)	0.0056	773:1231
EMA	82	1.3 (0.1–3.4)	1169	10	3.2 (1.4–7.2)	0.009	1048:2543
Biopsy	-	-	-	-	-	-	-
**RSA**							
Anti-TG2	69	2.2 (0.6–4.8)	1046	0	4.4 (1.5–12.7)	0.005	338:546
EMA	76	1.1 (0–3.9)	928	58 **	1.9 (0.1–25.8)	0.63	230:421
Biopsy	-	-	-	-	-	-	-

Only studies with sample sizes>100 individuals were included. For I^2^ ≥ 25%, a random model has been used. TG2: type 2 transglutaminase; EMA: antiendomisium antibodies; RSA: recurrent spontaneous abortion. * After excluding Vancikova et al. [10]; ** only two studies were included.
